# Establishing disease causality for a novel gene variant in familial dilated cardiomyopathy using a functional in-vitro assay of regulated thin filaments and human cardiac myosin

**DOI:** 10.1186/s12881-015-0243-5

**Published:** 2015-10-26

**Authors:** Stephen Pan, Ruth F. Sommese, Karim I. Sallam, Suman Nag, Shirley Sutton, Susan M. Miller, James A. Spudich, Kathleen M. Ruppel, Euan A. Ashley

**Affiliations:** Leon H. Charney Division of Cardiology, NYU Langone Medical Center, New York, NY USA; Department of Genetics, Cell Biology, and Development, University of Minnesota, Minneapolis, MN USA; Departments of Medicine (Cardiovascular Medicine), Stanford University School of Medicine, Stanford, CA USA; Department of Biochemistry, Stanford University School of Medicine, Stanford, CA USA; Department of Pediatrics (Cardiology), Stanford University School of Medicine, Stanford, CA USA; Department of Pharmaceutical Chemistry, University of California San Francisco, San Francisco, CA USA

**Keywords:** Cardiomyopathy, Troponin T, Myosin, Calcium, Gene mutation

## Abstract

**Background:**

As next generation sequencing for the genetic diagnosis of cardiovascular disorders becomes more widely used, establishing causality for putative disease causing variants becomes increasingly relevant. Diseases of the cardiac sarcomere provide a particular challenge in this regard because of the complexity of assaying the effect of genetic variants in human cardiac contractile proteins.

**Results:**

In this study we identified a novel variant R205Q in the cardiac troponin T gene (*TNNT2*). Carriers of the variant allele exhibited increased chamber volumes associated with decreased left ventricular ejection fraction. To clarify the causal role of this variant, we generated recombinant variant human protein and examined its calcium kinetics as well as the maximally activated ADP release of human β-cardiac myosin with regulated thin filaments containing the mutant troponin T. We found that the R205Q mutation significantly decreased the calcium sensitivity of the thin filament by altering the effective calcium dissociation kinetics.

**Conclusions:**

The development of moderate throughput post-genomic assays is an essential step in the realization of the potential of next generation sequencing. Although technically challenging, biochemical and functional assays of human cardiac contractile proteins of the thin filament can be achieved and provide an orthogonal source of information to inform the question of causality for individual variants.

## Background

Dilated cardiomyopathy (DCM) is characterized by thinning of the heart walls, enlargement of the left ventricular chamber, and weakness of the cardiac muscle resulting in an inability to raise the cardiac output to meet the demands of even modest exercise. DCM can result from many causes but a significant portion of cases are likely familial, with some estimates implicating a genetic predisposition in up to 30–40 % of cases of idiopathic DCM [1]. Identifying the genetic basis in these cases has proven challenging and there seems to be marked locus heterogeneity, with more than 50 different genes thus far implicated [2, 3]. In the context of next generation sequencing (NGS), this presents a particular challenge in distinguishing benign DNA variants from those that are clinically significant due to the large number of variants that are usually found with NGS [4]. In addition, establishment of co-segregation within families is complicated by marked variation in expressivity among family members, something that also implies environmental and genetic modifiers. Since sequencing for genetic diagnosis is increasingly available and as most genetic variation is very rare or private [5], there is an urgent need for functional assays of the human cardiac sarcomeric proteins to help establish causal contributions of novel variants. This is particularly relevant in DCM since currently available medical therapy can halt the progression of disease in many patients and their at-risk family members.

**Fig. 1:**
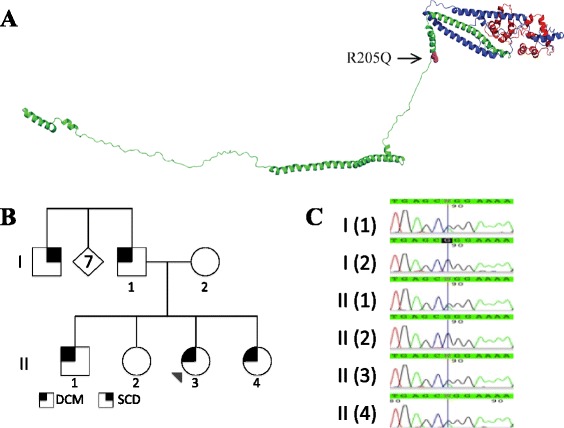
Identification of the novel R205Q troponin T mutation. **a** A model of the troponin complex, built from partial crystal structures of the human cardiac troponin complex of TnT (green), TnI (blue), and TnC (red) (Adapted from [22], PDB 1J1E). The R205Q mutation is shown in purple. **b** Pedigree for the family studied demonstrating inheritance of *TNNT2* mutation. Squares denote males, circles females, and the diamond denotes multiple family members (7 in total) for brevity. Arrow denotes the proband. **c** Raw chromatogram data from the sequencing of the *TNNT2* variant in multiple family members. DCM = dilated cardiomyopathy. SCD = sudden cardiac death

In this study, we describe a family with familial DCM for which a novel candidate variant (R205Q) was identified in the cardiac sarcomeric protein troponin T (Fig. 1a). Troponin T (TnT), along with troponin C (TnC) and troponin I (TnI), comprise the troponin complex, which acts as a Ca^2+^ sensor in the sarcomere and regulates the interaction of cardiac myosin with actin. Using expressed recombinant human proteins, we show that the candidate variant significantly decreases the calcium sensitivity of the thin filament — a hallmark of DCM-causing variants in thin filament proteins. Thus, we demonstrate the role of biochemical assays in clarifying the pathophysiological basis of disease in a family with a novel candidate variant.

## Results

### Molecular genetic data

We recruited a family of Hispanic descent with multiple family members affected by DCM (Fig. 1b). The proband was diagnosed with severe decompensated cardiomyopathy at age 4 and subsequently underwent cardiac transplantation. Her family history was notable for a paternal uncle who died at a young age of sudden cardiac death, which led to the consideration of familial DCM as a possible diagnosis. The proband was screened with commercial genetic testing (GeneDx) comprised of the sequencing of the coding exons of 23 genes known to be associated with DCM (full list below). The results were notable for a variant in the cardiac troponin T gene (*TNNT2*) located in exon 13 (NM_001001430.1 c.614 C > T, p.R205Q). This variant has not been reported previously in the literature. Although there have been previously reported associations of DCM with variants at this position, the nature of this novel variant was unknown [4, 6]. This variant was verified independently via Sanger sequencing in the proband. Further genotyping of the nuclear family revealed that the variant was also found in the father and two siblings of the proband. The mother and one sibling did not carry the variant (Fig. 1b, c). No other rare (allele frequency < 1 %) non-synonymous variants were found in the proband’s sequenced cardiac genes.

This variant has not previously been observed in hundreds of patients who have undergone sarcomeric gene sequencing amongst the clinical cohort at the Stanford Center for Inherited Cardiovascular Disease, of which a significant minority are also of Hispanic descent. Additionally, it was also not found in the 6503 exomes publicly available in the National Heart, Lung, and Blood Institute’s (NHLBI) Exome Variant Server from the NHLBI Grand Opportunity Exome Sequencing Project [7], although this dataset consists primarily of subjects of European-American and African-American descent. *In silico* tools (SIFT and polyphen-2) both predicted that this variant is likely to be pathogenic [8, 9]. The residue affected by this variant is highly evolutionarily conserved (GERP score of 4.440) [10].

### Clinical data

**Fig. 2:**
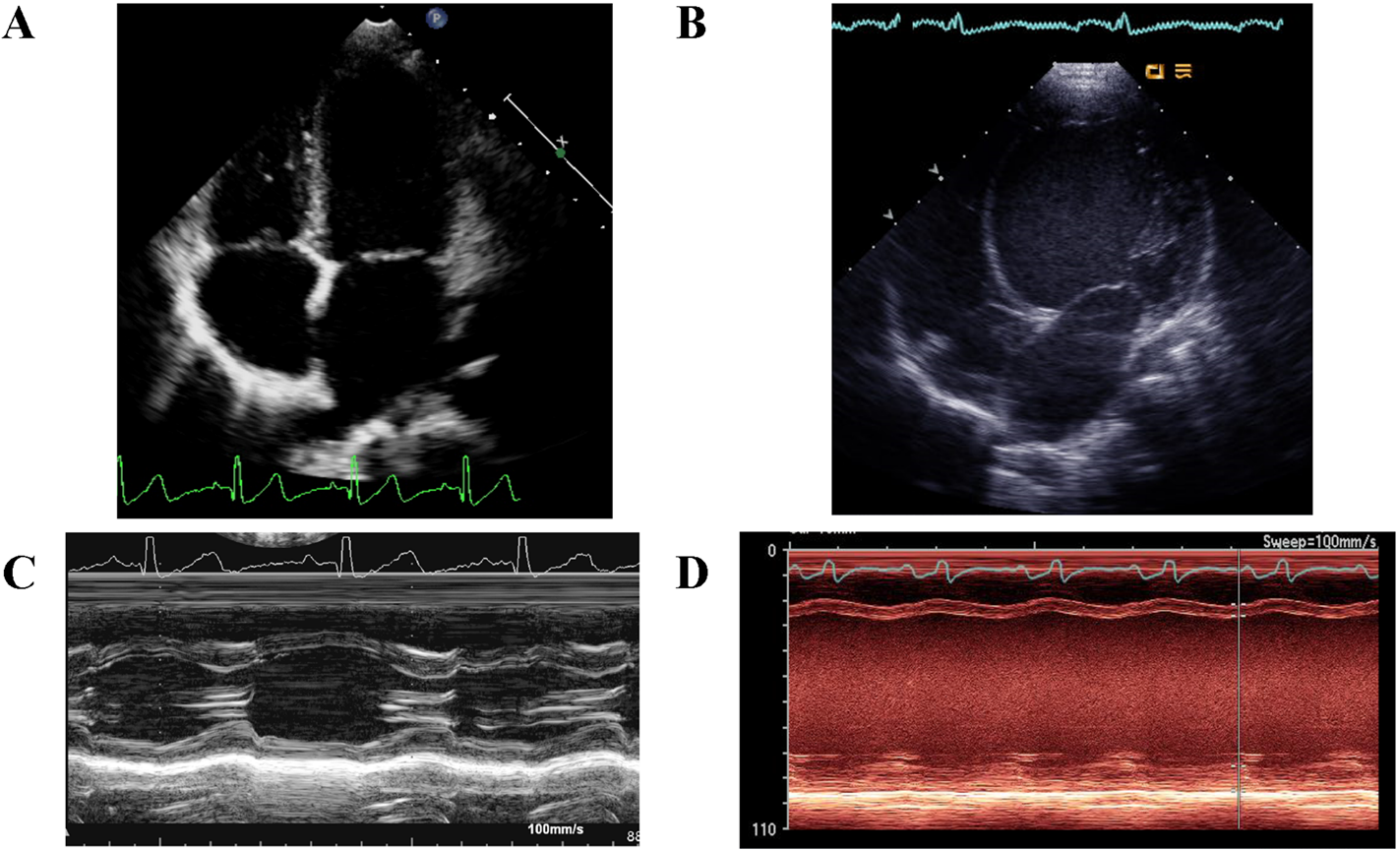
Transthoracic echocardiographic 4 chamber views (**a**, **b**) and m-mode at the level of the mitral valve (**c**, **d**) of the II-2 unaffected sibling (**a**, **c**) and the II-3 proband (**b**, **d**). A severely dilated left ventricle with poor systolic function is noted in the proband, while the unaffected sibling has normal cavity size and normal systolic function

Clinical disease was not evident in any family member other than the proband prior to clinical screening. Transthoracic two dimensional echocardiographic analysis was performed on each of the family members. In addition to the proband (Fig. 2b and d), the two siblings also carrying the R205Q *TNNT2* mutation (one male, one female) showed evidence of marked left ventricular dilation (left ventricular internal diameter in diastole (LVIDd) of 4.4 – 6.6 cm) and reduced ejection fraction (13 – 47 %) (Table 1). This was also noted in the proband’s father (genotype positive), albeit his degree of ventricular dilation and dysfunction was less pronounced. The proband’s mother and other female sibling, both genotype negative, displayed normal left ventricular cavity size and ejection fraction (Table 1 and Fig. 2a and c). Thus, within this nuclear family, only those with the *TNNT2* R205Q variant showed evidence of cardiac systolic dysfunction. Indices of diastolic function (assessed by mitral inflow E/A ratio and mitral valve annulus lateral E peak velocity) were within normal limits for all family members.

**Table 1:** Parameters of systolic and diastolic function from transthoracic echocardiography from members of a family affected by DCM with the *TNNT2* R205Q variant

	Proband	Brother	Sister	Sister	Father	Mother
	II-3	II-1	II-4	II-2	I-1	I-2
Age at Time of Screening (years)	4	2	8	3	36	34
Presence of R205Q variant	Yes	Yes	Yes	No	Yes	No
Ejection Fraction (%)	13	47	37	64	38	57
LVIDd (cm)	6.6	4.4	6.4	3.7	5.1	4.5
Mitral Valve E/A	1.73	1.9	2.9	1.8	1.4	1.9
Lateral E’ velocity (cm s^−1^)	10	26	22	16	15.7	12
Lateral A’ velocity (cm s^−1^)	3	6	6	5	9.7	7

LVIDd = left ventricular internal dimension in diastole

### Functional data

**Fig. 3:**
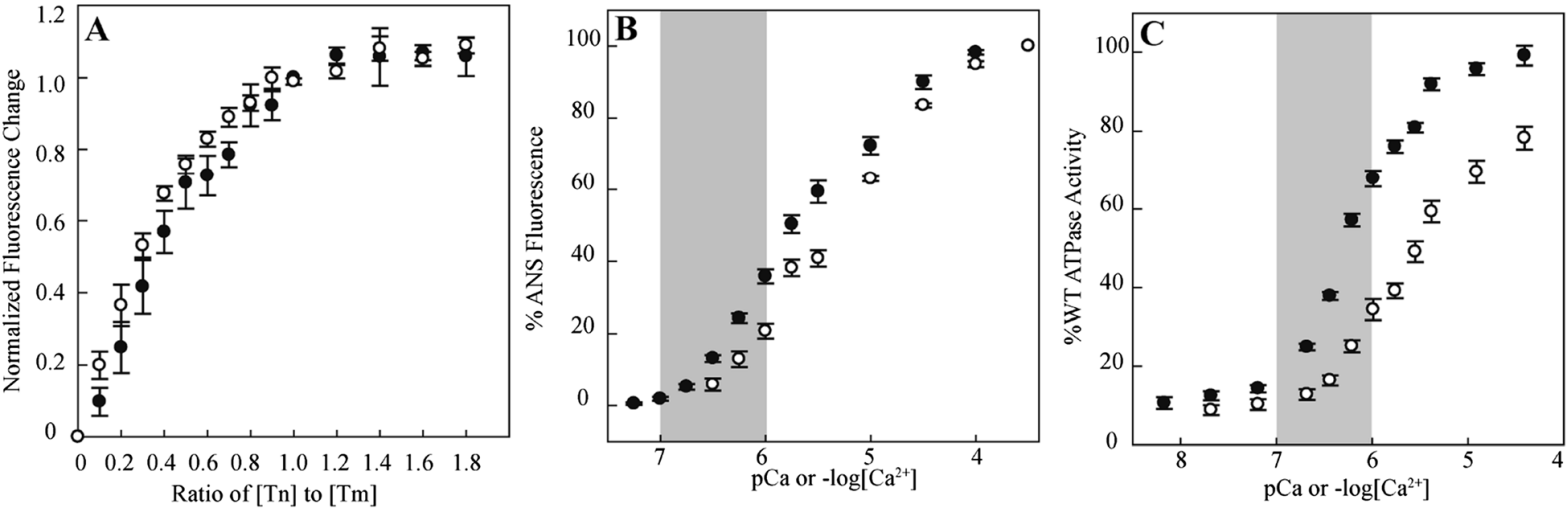
Tropomyosin binding and steady state WT and R205Q thin filament pCa curves. **a** Binding of WT (closed circles) and R205Q (open circles) troponin complex to pyrene-labeled tropomyosin. **b** Calcium sensitivity of the ANS-labeled thin filament in the absence of S1 at 23 °C. **c** Calcium sensitivity of thin-filament-activated ATPase activity of human β-cardiac myosin S1 at 30 °C. For both (**b**) and (**c**), each curve is the average of >10 individual curves, and error bars represent SEM. Each curve was individually fit by the Hill equation, as summarized in Table 2. The shaded gray region represents the average physiological Ca^2+^ range during a heartbeat [23, 24]

Troponin T (TnT), complexed with troponin I (TnI) and troponin C (TnC), binds to tropomyosin on the actin filament. We therefore first examined whether the R205Q mutation in the *TNNT2* gene alters the binding of the troponin complex to tropomyosin (Fig. 3a). The R205Q troponin complex appeared to have a slightly lower dissociation constant or K_d_ (48 ± 6 nM) compared to wild type (WT) (68 ± 6 nM), suggesting moderately tighter binding to tropomyosin for the mutant complex.

As previous work has shown that DCM-causing mutations in the troponin complex decrease the Ca^2+^ sensitivity of the thin filament [11, 12], we next examined the inherent Ca^2+^ binding properties of R205Q mutant thin filaments. We used a troponin complex containing a T53C modified troponin C (TnC) subunit (see Methods) which can be labeled with a hydrophobic fluorescent ANS probe [13]. Upon Ca^2+^ binding to the TnC subunit of the troponin complex, there is a conformational change in the troponin complex, which alters the environmentally sensitive ANS fluorescence signal (Fig. 3b). This signal change is a readout of the effective Ca^2+^ sensitivity of the complex and thin filament. Measuring this fluorescence signal as a function of increasing Ca^2+^ concentrations generates Ca^2+^ sensitivity, or pCa, curves. From these curves one can measure the Ca^2+^ concentration at which the thin filament is half-maximally activated (the pCa_50_) and determine an ‘effective dissociation constant’ ${K}_d={10}^{-pC{a}_{50}}$.

For both the WT and R205Q mutant troponin complexes alone, there was no difference in the effective K_d_ (7 ± 3 nM). With addition of actin and tropomyosin, however, the R205Q thin filament (4.4 ± 0.5 μM) showed a significant increase in the effective K_d_ compared to WT (1.2 ± 0.4 μM, *p* < 0.001), indicating a decrease in the Ca^2+^ sensitivity. A change in the Ca^2+^ sensitivity (K_d_) of the thin filament could reflect either an altered Ca^2+^ dissociation rate (*k*_*off*_) and/or Ca^2+^ association rate (*k*_*on*_). As Ca^2+^ binding is a very rapid process, it has generally been assumed that changes in the K_d_ for troponin mutations are mainly due to changes in *k*_*off*_ for the thin filament [14]. By stopped flow transient kinetic measurements, the effective *k*_*off*_ for the R205Q mutant thin filaments (177 ± 3 s^−1^) was indeed significantly higher than for the WT thin filaments (118 ± 1 s^−1^, *p* < 0.001), which is consistent with a higher K_d_ (Table 2).

**Table 2:** Summary of WT and R205Q mutant thin filament functional data with and without human β-cardiac myosin S1

	WT	R205Q
Effective K_d_ (μM)	1.2 ± 0.4	4.4 ± 0.5^*^
Effective *k*_*off*_ (s^−1^) at 23 °C	118 ± 1	177 ± 3^*^
S1 ATPase pCa50	6.17 ± 0.01	5.70 ± 0.02^*^
S1 ATPase ∆pCa50	------	−0.47
% S1 Maximum Activity	100 ± 1 %	80 ± 3 %^*^
% S1 Minimum Activity	10 ± 1 %	8 ± 1 %
n_H_ (S1 ATPase pCa Curve)	1.2 ± 0.1	1.1 ± 0.1
S1 ADP Release at 23 °C (s^−1^)	68 ± 1	67 ± 1
Tropomyosin binding, K_d_ (nM)	68 ± 6	48 ± 6

Means ± SEM

*n* represents: (1) for ANS, number of individual curves; ≥ 11 for K_d_; ≥ 9 for *k*_*off*_: (2) for ATPase, number of individual pCa Curves; 33, WT; 11, R205Q; (3) for ADP release, number of individual curves; 8, WT; 6, R205Q; (4) for tropomyosin binding, number of individual curves, ≥ 3

^*^ *p* < 0.001 by Student *t*-test or if unequal variance, the Mann-Whitney rank sum test

As tropomyosin and troponin regulate the binding and kinetics of the myosin-actin interaction in cardiac muscle [15, 16], we next examined the effect of the R205Q mutation in the presence of human β-cardiac myosin. Using the thin filament-activated myosin ATPase assay and a truncated single-headed human β-cardiac myosin (S1), we measured the Ca^2+^-sensitivity of the thin filament-myosin interaction. As with the ANS assay, thin filaments containing the R205Q variant TnT demonstrated a decreased Ca^2+^ sensitivity, with a change in the pCa_50_ of −0.47. Interestingly, there was also a ~20 % decrease in the maximal activation of the thin filament which could reflect a slight decrease in affinity of the myosin for the R205Q thin filaments (Table 2, Fig. 3c).

An additional functional parameter that has been suggested to be altered by DCM-causing troponin mutations is the maximally activated ADP release rate of β-cardiac myosin. For β-cardiac myosin, the ADP release rate determines the amount of time a myosin molecule will remain strongly bound to actin and is therefore a key parameter in contractile function and force production [16, 17]. Using human β-cardiac myosin S1, we measured the maximal Ca^2+^-activated ADP-release by stopped flow. We observed no significant change in the maximal ADP-release between WT and R205Q thin filaments with both ~67 s^−1^ (Table 2). This is in agreement with a previous study on cardiomyopathy-causing thin filament variants using human β-cardiac myosin, which similarly demonstrated that the inherent actomyosin ADP release kinetics at saturating Ca2+ concentrations were not altered [11].

Overall, clear differences between the functional kinetics of WT and R205Q thin filaments were seen. The R205Q thin filaments exhibited decreased Ca^2+^ sensitivity, attributable to a decreased rate of Ca^2+^ dissociation, in both the presence and absence of β-cardiac myosin. For DCM-causing mutations in the troponin complex, changes in the pCa_50_ generally range from −0.1 to −0.5 [18]. Thus, this mutation appears to cause a significant decrease in calcium sensitivity (Δ pCa_50_ =−0.47) that is at the severe end of the observed range of mutation-induced effects.

## Discussion

The advent of next generation sequencing technologies has revealed that most human genetic variation is rare. The wealth of genomic data now available presents both a great opportunity for genetic discovery but also a new challenge in the definition of variants that truly contribute to disease [5, 19–21]. In this study, we took a novel variant R205Q in *TNNT2* in a family with DCM, and using co-segregation, clinical data, and correlating biochemical studies, present a case for pathogenicity. From the biochemical studies, we found that the DCM-causing R205Q *TNNT2* variant primarily altered the Ca^2+^ kinetics of the thin filament, significantly decreasing the overall effective Ca^2+^ sensitivity, a trend that is consistent with other DCM-causing troponin mutations [12]. The residue affected by this variant is located in the linker region of TnT, which connects the N-terminal tropomyosin-binding portion of TnT to the C-terminal TnI-TnC binding region (Fig. 1). According to the three state model for thin filament activation, upon Ca^2+^ binding, TnC undergoes a conformational change that results in the dissociation of the TnI inhibitory subunit. This dissociation allows tropomyosin to azimuthally shift, exposing the myosin binding sites. The TnT linker region is important in communicating these changes in the actin-tropomyosin region to the TnI-TnC region and vice-versa. Thus it is not surprising that changes in the amino acid structure in this region could lead to the observed functional effects [22].

During contraction, the Ca^2+^ levels in the sarcomere of the cardiomyocyte increase from ~10^−7^ to 10^−6^ M (grey region in Fig. 3) [23, 24]. A decrease in the Ca^2+^ sensitivity significantly lowers the number of force-producing myosin cross-bridges and reduces overall systolic force production [25], as is seen in DCM patients. At [Ca^2+^] levels below ~10^−7^ M, such as seen in diastole, there was no major difference in the Ca^2+^sensitivity of the mutant filament. These biochemical parameters mirrored the clinical parameters in members of this family with this variant, with reduced systolic function as measured by ejection fraction and chamber size, and normal parameters of diastolic function including the E/A ratio and E’ velocity.

Previous work has explored the use of functional assays of thin filament proteins to evaluate the effects of mutations. For example, Hershberger et al [4] used a model incorporating mutant TnT into porcine-skinned cardiac fiber preparations using fiber tension as a readout of calcium sensitivity with excellent results. Our work builds on this by using an entirely in-vitro system incorporating both human troponin subunits and human *β-*cardiac myosin to directly assess Ca^2+^ sensitivity. This biochemical approach allows for a rapid, moderate throughput screening of human genetic variants in these proteins to provide evidence for or against pathogenicity. We successfully were able to use this approach to find a significant change in the Ca^2+^ sensitivity for the R205Q variant TnT reported here, which combined with the genetic and clinical data, identifies R205Q as a novel DCM-causing mutation.

This model does have some limitations. Although the model incorporates human troponin and myosin, other sarcomeric proteins such as titin, myosin binding protein C, and others are not incorporated into this in-vitro model and thus it cannot inform on the effects of variants in these other genes. Also, as noted before, the readout of this model is based on the calcium sensitivity of the thin filament-myosin complex. If a variant’s pathogenicity is through another mechanism, this model would not apply. In this particular family case, we cannot rule out contributions to pathogenicity from variants in other genes that were not sequenced in the original panel, including genes that have more recently been implicated in the pathogenicity of DCM such as titin or BAG3. If such modifier variants did exist this model could not assess the relative contributions from these other variants, although it could be used to assess relative contributions from multiple variants in the thin filament and myosin.

## Conclusions

The development of moderate throughput post-genomic assays is an essential step in the realization of the potential of next generation sequencing. Although technically challenging, biochemical and functional assays of the cardiac regulated thin filaments and human cardiac myosin can be achieved and provide an orthogonal source of information to inform the question of causality for individual variants.

## Methods

### Ethics

All research involving human subjects (including human material and human data) was performed in accordance with the Declaration of Helsinki. The Institutional Review Board at Stanford University approved the study. Written informed consent was obtained from all adult participating subjects, specifically the mother and father of the proband. For their children, including the proband, all of whom were minors at the time of the study, informed consent was obtained from the parents as well.

### Patient clinical evaluation

All nuclear family members underwent transthoracic echocardiographic screening to evaluate cardiac function. Standardized measurements of cardiac chamber size and volume, valvular function, and systolic and diastolic function were carried out according to American Society of Echocardiography guidelines.

### Genetic testing

Genetic testing of the proband was performed by sequencing 23 genes previously associated with DCM: *LMNA*, *LDB3*, *TNNT2*, *DES*, *SGCD*, *PLN*, *ACTC1*, *MYH7*, *TPM1*, *TNNI3*, *TAZ*, *TTR*, *MYBPC3*, *LAMP2*, *MTTK*, *MTTL1*, *MTTQ*, *MTTH*, *MTTS1*, *MTTS2*, *MTND1*, *MTND5*, and *MTND6* (GeneDx). Targeted PCR and Sanger sequencing of exon 13 of TNNT2 was carried out in the proband and 5 immediate nuclear family members to confirm the presence or absence of the variant. Blood samples were obtained and DNA was isolated from buffy coat samples (Epicentre, MasterPure™ DNA Purification Kit). The primers used were as follows: forward, 5′-CAGGGGGTTTGGGGAGGGTTAG-3′; reverse, 3′-GTGGGGCACCTGCTCAGTTCTCT-5′.

### Cloning of human cardiac troponin mutants and human β-cardiac myosin

The R205Q mutation was introduced by QuikChange site-directed mutagenesis (Stratagene) into human adult cardiac TnT (*TNNT2*) and confirmed by sequencing. For fluorescence experiments, a similar strategy was used to make the triple mutation of TnC containing C35S, T53C, and C84S. A truncated version of human cardiac myosin heavy chain 7 (*MYY7*) corresponding to a short S1 (residues 1–808), with (for ATPase and motility measurements) and without (for stop flow measurements) an eGFP linker, was constructed and produced as previously described [11, 26].

### Protein expression

Human adult cardiac troponin subunit (*TNNT2*, *TNNI3*, *TNNC2*) expression and purification methods were based on previously published protocols [27–30]. Tropomyosin was purified from bovine cardiac tissue according to the protocol of Smillie [31]. Chicken skeletal actin and human β-cardiac myosin were prepared as previously described [26].

### Tropomyosin pyrene binding

Tropomyosin pyrene labeling and binding assays were performed as described previously [11]. All binding experiments were carried out with 100 nM pyrene-tropomyosin at 23 °C in 20 mM Hepes, pH 7.5, 100 mM KCl, 2 mM MgCl_2_, and 1 mM DTT.

### Coupled actin-activated ATPase assay

We used the NADH-coupled assay at 30 °C to measure the Ca^2+^ sensitivity of the steady state actin-activated ATPase rate for the human β-cardiac S1 [11, 32]. The concentrations of freshly prepared proteins used were 3.5 μM actin, 1 μM tropomyosin, 2 μM troponin, and 0.3 to 0.5 μM S1. The final buffer conditions were 20 mM imidazole, 10 mM KCl, 2 mM MgCl_2_, 1 mM DTT, 2 mM ATP, the Ca^2+^ buffers (2 mM EGTA, 4 mM NTA, and varying concentrations of CaCl_2_), and the NADH-coupling system [32]. The pCa Calculator developed by Dweck et al. was used to determine the composition of the Ca^2+^ buffers with EGTA and NTA [33].

### ANS troponin complex

The triple TnC mutant was labeled with the fluorescent dye IAANS and ANS-labeled TnC steady state assays were performed in a Fluorolog fluorimeter (Horiba Scientific) at 23 °C, similar to previous studies [11, 13, 34]. Final buffer conditions were 200 mM Hepes, pH 7.5, 10 mM KCl, 3 mM MgCl_2_, 1 mM DTT, 4 mM NTA, and 2 mM EGTA, and the amount of CaCl_2_ added was determined using the pCa calculator [33]. Transient Ca^2+^ dissociation rates were measured using a HiTech SF-61DX2 (TgK Scientific Ltd., U.K.) stopped-flow apparatus at 23 °C as previously described [11]. Each data trace was individually fit to a single exponential function, with more than five individual traces for both WT and R205Q.

### ADP release

Actin was labeled similarly to previous methods [35]. ADP release rates were measured using a HiTech SF-61DX2 (TgK Scientific Ltd., U.K.) stopped-flow apparatus at 23 °C in 25 mM Hepes, pH 7.5, 25 mM KCl, 4 mM MgCl_2_, 0.2 mM CaCl_2_, and 1 mM DTT. For WT and R205Q thin filaments, ≥6 traces were collected and fit individually. Regulated thin filaments were prepared with S1-ADP at a final concentration of 2 μM actin, 0.5 μM tropomyosin, 0.5 μM troponin, 2 μM S1, and 50 μM ADP and then rapidly mixed with 2 mM ATP, 50 μM ADP.

## References

[1] Hershberger RE, Morales A, Siegfried JD (2010). Clinical and genetic issues in dilated cardiomyopathy: a review for genetics professionals. Genet Med.

[2] Lakdawala NK, Funke BH, Baxter S, Cirino AL, Roberts AE, Judge DP (2012). Genetic testing for dilated cardiomyopathy in clinical practice. J Card Fail.

[3] McNally EM, Golbus JR, Puckelwartz MJ (2013). Genetic mutations and mechanisms in dilated cardiomyopathy. J Clin Invest.

[4] Hershberger RE, Pinto JR, Parks SB, Kushner JD, Li D, Ludwigsen S (2009). Clinical and functional characterization of TNNT2 mutations identified in patients with dilated cardiomyopathy. Circ Cardiovasc Genet.

[5] Pan S, Caleshu CA, Dunn KE, Foti MJ, Moran MK, Soyinka O (2012). Cardiac structural and sarcomere genes associated with cardiomyopathy exhibit marked intolerance of genetic variation. Circ Cardiovasc Genet.

[6] Mogensen J, Murphy RT, Shaw T, Bahl A, Redwood C, Watkins H (2004). Severe disease expression of cardiac troponin C and T mutations in patients with idiopathic dilated cardiomyopathy. J Am Coll Cardiol.

[7] Exome Variant Server [http://evs.gs.washington.edu/EVS/]. Accessed January 20th, 2014.

[8] Kumar P, Henikoff S, Ng PC (2009). Predicting the effects of coding non-synonymous variants on protein function using the SIFT algorithm. Nat Protoc.

[9] Adzhubei IA, Schmidt S, Peshkin L, Ramensky VE, Gerasimova A, Bork P (2010). A method and server for predicting damaging missense mutations. Nat Methods.

[10] Cooper GM (2005). Distribution and intensity of constraint in mammalian genomic sequence. Genome Res.

[11] Sommese RF, Nag S, Sutton S, Miller SM, Spudich JA, Ruppel KM (2013). Effects of troponin T cardiomyopathy mutations on the calcium sensitivity of the regulated thin filament and the actomyosin cross-bridge kinetics of human β-cardiac myosin. PLoS ONE.

[12] Willott RH, Gomes AV, Chang AN, Parvatiyar MS, Pinto JR, Potter JD (2010). Mutations in Troponin that cause HCM, DCM AND RCM: what can we learn about thin filament function?. J Mol Cell Cardiol.

[13] Davis JP, Norman C, Kobayashi T, Solaro RJ, Swartz DR, Tikunova SB (2007). Effects of thin and thick filament proteins on calcium binding and exchange with cardiac troponin C. Biophys J.

[14] Davis JP, Tikunova SB (2008). Ca(2+) exchange with troponin C and cardiac muscle dynamics. Cardiovasc Res.

[15] McKillop DF, Geeves MA (1993). Regulation of the interaction between actin and myosin subfragment 1: evidence for three states of the thin filament. Biophys J.

[16] Gordon AM, Homsher E, Regnier M (2000). Regulation of contraction in striated muscle. Physiol Rev.

[17] Deacon JC, Bloemink MJ, Rezavandi H, Geeves MA, Leinwand LA (2012). Erratum to: Identification of functional differences between recombinant human α and β cardiac myosin motors. Cell Mol Life Sci.

[18] Mirza M, Marston S, Willott R, Ashley C, Mogensen J, McKenna W (2005). Dilated cardiomyopathy mutations in three thin filament regulatory proteins result in a common functional phenotype. J Biol Chem.

[19] Norton N, Robertson PD, Rieder MJ, Züchner S, Rampersaud E, Martin E (2012). Evaluating pathogenicity of rare variants from dilated cardiomyopathy in the exome era. Circ Cardiovasc Genet.

[20] Jabbari J, Jabbari R, Nielsen MW, Holst AG, Nielsen JB, Haunsø S (2013). New exome data question the pathogenicity of genetic variants previously associated with catecholaminergic polymorphic ventricular tachycardia. Circ Cardiovasc Genet.

[21] Andreasen C, Nielsen JB, Refsgaard L, Holst AG, Christensen AH, Andreasen L (2013). New population-based exome data are questioning the pathogenicity of previously cardiomyopathy-associated genetic variants. Eur J Hum Genet.

[22] Manning EP, Tardiff JC, Schwartz SD (2011). A model of calcium activation of the cardiac thin filament. Biochemistry.

[23] Stehle R, Iorga B (2010). Kinetics of cardiac sarcomeric processes and rate-limiting steps in contraction and relaxation. J Mol Cell Cardiol.

[24] McDonald KS, Herron TJ (2002). It takes “heart” to win: what makes the heart powerful?. News Physiol Sci.

[25] Lakdawala NK, Thune JJ, Colan SD, Cirino AL, Farrohi F, Rivero J (2012). Subtle abnormalities in contractile function are an early manifestation of sarcomere mutations in dilated cardiomyopathy. Circ Cardiovasc Genet.

[26] Sommese RF, Sung J, Nag S, Sutton S, Deacon JC, Choe E (2013). Molecular consequences of the R453C hypertrophic cardiomyopathy mutation on human β-cardiac myosin motor function. Proc Natl Acad Sci.

[27] Szczesna D, Zhang R, Zhao J, Jones M, Guzman G, Potter JD (2000). Altered regulation of cardiac muscle contraction by troponin T mutations that cause familial hypertrophic cardiomyopathy. J Biol Chem.

[28] Pan BS, Potter JD (1992). Two genetically expressed troponin T fragments representing alpha and beta isoforms exhibit functional differences. J Biol Chem.

[29] Sheng Z, Pan BS, Miller TE, Potter JD (1992). Isolation, expression, and mutation of a rabbit skeletal muscle cDNA clone for troponin I. The role of the NH2 terminus of fast skeletal muscle troponin I in its biological activity. J Biol Chem.

[30] Szczesna D, Guzman G, Miller T, Zhao J, Farokhi K, Ellemberger H (1996). The role of the four Ca2+ binding sites of troponin C in the regulation of skeletal muscle contraction. J Biol Chem.

[31] Smillie LB (1982). Preparation and identification of alpha- and beta-tropomyosins. Methods Enzymol.

[32] La Cruz De EM, Ostap EM (2009). Kinetic and equilibrium analysis of the myosin ATPase. Methods Enzymol.

[33] Dweck D, Reyes-Alfonso A, Potter JD (2005). Expanding the range of free calcium regulation in biological solutions. Anal Biochem.

[34] Liu B, Tikunova SB, Kline KP, Siddiqui JK, Davis JP (2012). Disease-related cardiac troponins alter thin filament Ca2+ association and dissociation rates. PLoS ONE.

[35] Kouyama T, Mihashi K (1981). Fluorimetry study of N-(1-pyrenyl)iodoacetamide-labelled F-actin. Local structural change of actin protomer both on polymerization and on binding of heavy meromyosin. Eur J Biochem.

